# A multiple testing procedure for multi-dimensional pairwise comparisons with application to gene expression studies

**DOI:** 10.1186/s12859-016-0937-5

**Published:** 2016-02-25

**Authors:** Anjana Grandhi, Wenge Guo, Shyamal D. Peddada

**Affiliations:** BARDS, Merck Research Laboratories, RY34-A3086h, 126 E. Lincoln Avenue, Rahway, 07065 NJ USA; Department of Mathematical Sciences, New Jersey Institute of Technology, Newark, 07102 NJ USA; Biostatistics and Computational Biology Branch, National Institute of Environmental Health Sciences, T.W.Alexander Drive, Research Triangle Park, 27709 NC USA

**Keywords:** mdFDR, BH procedure, Multiple testing, Differential gene expression, Tumor size

## Abstract

**Background:**

Often researchers are interested in comparing multiple experimental groups (e.g. tumor size) with a reference group (e.g. normal tissue) on the basis of thousands of features (e.g. genes) and determine if a differentially expressed feature is up or down regulated in a pairwise comparison. There are two sources of false discoveries, one due to multiple testing involving several pairwise comparisons and the second due to falsely declaring a feature to be up (or down) regulated when it is not (known as directional error). Together, the total error rate is called the mixed directional false discovery rate (mdFDR).

**Results:**

We develop a general powerful mdFDR controlling testing procedure and illustrate the methodology by analyzing uterine fibroid gene expression data (PLoS ONE 8:63909, 2013). We identify several differentially expressed genes (DEGs) and pathways that are specifically enriched according to the size of a uterine fibroid.

**Conclusions:**

The proposed general procedure strongly controls mdFDR. Several specific methodologies can be derived from this general methodology by using appropriate testing procedures at different steps of the general procedure. Thus we are providing a general framework for making multiple pairwise comparisons. Our analysis of the uterine fibroid growth gene expression data suggests that molecular characteristics of a fibroid changes with size. Our powerful methodology allowed us to draw several interesting conclusions regarding the molecular characteristics of uterine fibroids. For example, IL-1 signaling pathway (Sci STKE 2003:3, 2003), associated with inflammation and known to activate prostaglandins that are implicated in the progression of fibroids, is significantly enriched only in small tumors (volume < 5.7 *cm*^3^). It appears that the molecular apparatus necessary for fibroid growth and development is established during tumor development. A complete list of all DEGs and the corresponding enriched pathways according to tumor size is provided for researchers to mine these data. Identification of these DEGs and the pathways may potentially have clinical implications.

**Electronic supplementary material:**

The online version of this article (doi:10.1186/s12859-016-0937-5) contains supplementary material, which is available to authorized users.

## Background

Increasingly it is a commonplace for researchers to conduct large scale genomic studies involving multiple experimental groups along with a control group, also called the normal or the reference group. The goal is to determine features that are differentially expressed in a given experimental group (relative to the reference group) and to determine if a differentially expressed feature is up or down regulated. For example, a toxicologist may be interested in identifying differences in the gene expression profile of spontaneous tumors and chemically induced tumors, relative to normal tissues [1–4]. There is considerable interest among cancer researchers to understand the gene expression profile of tumors according to tumor size [5–9]. Accordingly, this has been an active area of research for various cancers over the past decade [10, 11]. For example, Gieseg et al. [6] investigated gene expression of 3 different sizes of colon cancer tumors and found gene expressions to be constant with tumor size. However, Diaz et al. [5] demonstrated that the gene expression of *β*4 integrin is correlated with both size and nuclear grade of the breast cancer tumors, with increased expression in large tumors and in higher tumor grades. More recently Riis et al. [9] conducted an extensive gene expression analysis of breast cancer tumors according to size categories and identified genes and activation pathways that were specific to tumor size. They concluded that the molecular signatures derived from their analysis will help clinicians determine whether to treat tumors of a given size category aggressively or not. Earlier Ciarmela et al. [12] conducted an exhaustive study of the role of various growth factors on the development of uterine fibroids (leiomyoma). Thus it is clear there is considerable interest among clinicians and biologists to investigate the expression of genes according to the tumor size or category. In all such investigations, one is typically interested in performing several pairwise comparisons, of thousands of features, relative to a reference group (e.g. normal tissue). Often researchers are not only interested in determining if a feature is differentially expressed but are also interested in determining whether it is up or down-regulated in the experimental group (relative to the reference group). For simplicity of exposition, throughout this paper we shall replace the term “feature” by “gene”.

Multiple testing problems involving multiple pairwise comparisons of high dimensional data along with directional decisions has not received much attention in the literature, yet such testing problems are commonly encountered in practice. When the number of genes is very small (perhaps in tens) several methods have been proposed that control the directional errors as well as the family wise error rate [13–16]. However, such methods are very conservative when the number of genes is very large as in a microarray data or CpG methylation data [37–38]. Several ad-hoc methods and strategies are used in the literature when the number of genes is large. For example, some researchers apply multiple testing procedures (e.g. the Benjamini-Hochberg (BH) or Bonferroni procedure) within each pairwise comparison and ignore the fact that they are conducting several pairwise comparisons. Once a differentially expressed gene (DEG) for a pairwise comparison is identified then they are declared to be up or down-regulated by looking at the direction of the fold change (or the test statistic) without accounting for the statistical error associated with such a directional decision. Such strategies result in an inflated overall error rate due to multiple pairwise comparisons and directional decisions. This overall error rate is called the mixed directional false discover rate (mdFDR). Another procedure that is commonly used by researchers is to first perform ANOVA across all experimental groups for each feature. Using the resulting *p*-values, a multiple testing procedure is applied to obtain an initial list of DEGs. These DEGs are then subject to a Tukey’s test or a Bonferroni test to identify the pairwise comparisons where a DEG is significant. The directional decision regarding whether a gene is up or down regulated, is made on the basis of the direction of the fold change. While this strategy is more intuitive than the earlier one, it is potentially a conservative procedure resulting in a substantial loss of power, firstly because intrinsically ANOVA compares all treatment groups. Secondly, the thresholds or the critical values at the two steps are not derived with the goal to control the overall error rate.

The only formal methodology available in the literature that controls the mdFDR for the above directional multiple testing problems is the method by Guo et al. [17], which is designed to make decisions on thousands of features when making multiple pairwise comparisons and deciding on the direction of comparison. Thus the method controls the false discovery rate when making multiple pairwise comparisons on thousands of genes while also controlling the directional errors committed when falsely declaring a DEG to be up-regulated (or down-regulated) when it is not. Guo et al. [17] procedure generalizes the procedure of Benjamini and Yekutieli [18] which was designed for the case when there were only two groups to compare. The procedure of Guo et al. [17] is available in the software ORIOGEN (3.2) that can be accessed from http://www.niehs.nih.gov/research/atniehs/labs/bb/staff/peddada/.

While Guo et al. [17] methodology is useful for making several multiple pairwise comparisons; it is relatively conservative since it relies on the Bonferroni procedure to deal with multiple pairwise comparisons within each gene. In this paper we develop a general mdFDR controlling testing procedure that allows us to use any mixed directional familywise error rate (mdFWER) controlling procedure in place of the Bonferroni procedure, for conducting pairwise comparisons in high dimensional data that is broadly applicable to a wide range of genomic data including gene expression microarray data, CpG methylation data, RNA-seq data and others. Based on this general procedure, we develop a specific methodology using the Dunnett’s test [19–21] which is designed for making comparisons of several experimental groups with the control or the reference group. Not only that the resulting methodology is practically relevant but as demonstrated in the numerical simulations, the resulting methodology not only controls the mdFDR but is more powerful relative to some potential alternative methods.

We illustrate the proposed methodology by analyzing a gene expression data obtained from the NIEHS’ Fibroid Growth Study (FGS) [22]. Using our methodology we gain deeper insights into molecular characteristics of uterine fibroids according to the tumor size, which was not understood until now. We have identified several differentially expressed genes and pathways that are specifically enriched according to the tumor size. While researchers and clinicians who study fibroids are well aware of many of the genes and pathways described in this paper, we have provided a characterization of these genes and pathways according to the tumor size. Our data can be further mined to gain deeper insights regarding fibroids.

## Methods

In this section we present the general testing procedure that controls mdFDR while making multidimensional directional decisions, and develop the statistical methodology based on Dunnett’s test that is used to analyze FGS gene expression data. The relevant notations, definitions, hypotheses and other statistical concepts are described in the online Additional file 6 (see Sections S1 and S3) and Additional file 1: Figure S1.

**The general mdFDR controlling procedure**

**The general testing algorithm:**

1. Use any global testing method to obtain the screening *p*-values. Apply BH procedure [23] to identify genes that are differentially expressed in at least one pairwise comparison. Let *R* denote the number of genes so discovered.
2. For each gene discovered in step 1, use any two-sample testing method to obtain the *p*-values for each pairwise comparison and apply any mixed directional family wise error (mdFWER) controlling procedure, such as Holm, Hochberg etc. [24, 25], to the pairwise *p*-values at level *Rα*/*m*.
3. For a given gene discovered in step 1, if a pairwise hypothesis is rejected in step 2, then we declare the gene to be up or down regulated in the pairwise comparison according to the sign of the corresponding test statistic.

This procedure is general in the sense that any global testing method can be used to obtain the screening *p*-values in step 1 of the procedure, any pairwise comparison testing method can be used to obtain the pairwise *p*-values in step 2 and any mdFWER controlling procedure can be used in steps 2-3 of the procedure. It is important to note here that the method of Guo et al. [17] is a special case of the proposed general procedure in which Bonferroni global test is used for testing the screening hypotheses and Bonferroni method along with additional directional decisions works as the mdFWER controlling procedure. This general algorithm is proved to control mdFDR through the following theorem with the mathematical proof provided in the online Additional file 6: Section S2.

### **Theorem 1**.

Under assumption of independence of *p*-value vectors *P*_*j*_, *j*=1,…,*m*, the mdFDR of the General Testing Algorithm is strongly controlled at level *α*.

***Statistical methodology for FGS gene expression data***

Suppose we are interested in comparing “*q*” experimental groups with a reference group (in total *p*=*q*+1 groups) on the basis of the mean expression of “*m*” genes. For example, suppose we are interested in comparing “small”, “medium” and “large” fibroids with a “normal” tissue (also called normal myometrium) from uterus on the basis of “m” genes. Our goal is not only to identify differentially expressed genes in any given pairwise comparison but also to determine if the mean expression is up or down-regulated in the tumor tissue compared to the normal myometrium. Our statistical methodology for the FGS gene expression data analysis proceeds in three steps as follows. Specific details of implementation of each step are described in the online Additional file 6: Section S3.

1. For each gene we obtain a Dunnett [19–21] based screening *p*-value from all “*q*” pairwise comparisons with the reference group. This results in one *p*-value for each gene. Applying the BH procedure [23] on these screening *p*-values, we obtain genes that are differentially expressed in at least one pairwise comparison. Suppose we discover *R* genes in this step. Thus there are *R* genes which are differentially expressed in at least one pairwise comparison with the reference group.
2. For the *j*^*th*^ gene discovered in Step 1, we compute Dunnett’s *p*-value *P*_*ij*_ for each pairwise comparison *i*, *i*=1,2,…,*q*, with the reference group. If $P_{\textit {ij}} \leq \frac {R\alpha }{m}$ then we declare that the *i*^*th*^ pairwise comparison with the reference group is significant.
3. If a gene *j* is found to be significant in the *i*^*th*^ pairwise comparison with the reference group then we declare it to be up-regulated in the *i*^*th*^ group relative to the reference group if *T*_*ij*_>0, otherwise it is declared to be down-regulated. Here *T*_*ij*_ denotes the test statistic associated with the *j*^*th*^ gene in the *i*^*th*^ pairwise comparison.

### **Remarks**.

It is important to note in Steps 1 and 2 that it is possible for a gene to be differentially expressed on the basis of the screening p-value, yet it may not be significant in any of the pairwise comparisons. This happens when the BH-adjusted *p*-values are only marginally significant. In such cases we declare that gene to be not differentially expressed. We expect this phenomenon to occur with very small frequency.

## Results and discussion

In this section we present two sets of numerical results. First, the operating characteristics of the proposed procedure based on a simulation study are presented. Next, we apply the proposed methodology to a gene expression data obtained from the NIEHS’ FGS to identify tumor size specific DEGs.

### Numerical simulation and discussion

In this section we present the operating characteristics of the proposed procedure based on a simulation study. This study evaluates the power of the proposed Dunnett based methodology and compares its performance with some competing procedures, the details of which are given in the online Additional file 6: Section S4.

In our simulation we considered *p*=4 groups, the first three were taken to be experimental groups and the last group was taken to be the reference group. Thus all pairwise comparisons are made with the last group. Our simulated microarray chip consisted of *m*=1000 genes per chip with *n*=10 chips per group. As often done in simulation studies for microarray data [17, 26–28], we generated the expression of each gene in each chip using a normal distribution.

More precisely, for the *j*^*th*^ gene, *j*=1,2,…,1000, in the *s*^*th*^ sample, *s*=1,2,…,10 in the *g*^*th*^ group, *g*=1,2,3,4, we generated its expression $Z_{\textit {gj}}^{s}$ from a normal distribution with mean value $E\left (Z_{\textit {gj}}^{s} \right)\vspace *{-2pt} = \mu _{\textit {gj}}$ and variance $V \left (Z_{\textit {gj}}^{s} \right) = 1$. For the reference group (i.e. group 4) we set *μ*_4*j*_=0 for all *j*. To create the null data we set *μ*_*gj*_=0 for *j*=1,2,…,*m*_0_, *g*=1,2,3 and non-null data were created by generating *μ*_*gj*_∼^*independent*^*U*(0,2.5), for *g*=1,2,3 and *j*=*m*_0_+1,*m*_0_+2,…,*m*_0_+*m*_1_, where *m*_1_=*m*−*m*_0_ and *U* represents the uniform distribution. It is important to note that, for the non-null means considered here, the standard deviation used in this simulation study is large. Consequently, all methods considered in this simulation study are expected to have small power. We considered three patterns of correlation structure as follows: (a) Independent gene expressions: the correlation coefficient *ρ* between any pair of genes and any pair of sample is 0, thus, the data are completely independent. (b) Gene expressions within sample are dependent: for a given sample *s*, the correlation coefficient between any pair of genes is *ρ* but the correlation coefficient between any pair of samples is 0. (c) Gene expressions within genes are dependent: for a given gene *j*, the correlation coefficient between any pair of samples is *ρ* but the correlation coefficient between any pair of genes is 0. Although we considered a variety of patterns of *ρ*, due to space limitations we only present the results corresponding to independence. Similar results are obtained for other patterns of *ρ* (see the online Additional file 6: Section 4 for the results).

We simulated the mdFDR and average power using 1000 simulation runs. Our nominal mdFDR level was taken to be *α*=0.05. Details regarding the calculations of the mdFDR and power are provided in the accompanying online Additional file 6. For comparison purposes, we compared the proposed procedure with the Guo et al. [17] procedure and variants of the proposed procedure in which the mdFWER controlling procedures used in steps 2 and 3 of the algorithm are respectively, the Holm’s procedure [24], the Hochberg’s procedure [25] and the Bonferroni procedure. These procedures are described in detail in the online Additional file 6.

**Fig. 1:**
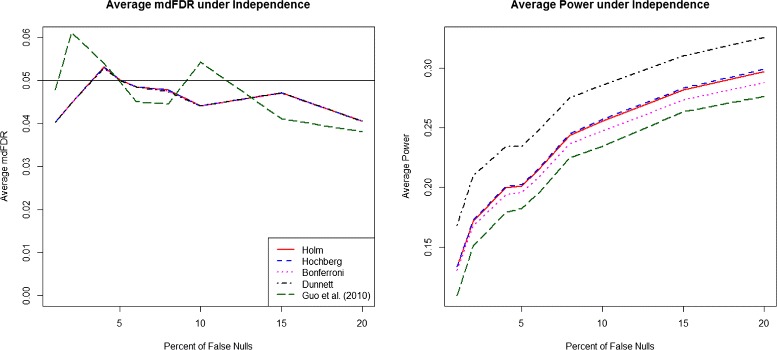
mdFDR (*left*), Average Power (*right*). mdFDR (*left*), Average Power (*right*) with the proposed methodology and three variants using Holm, Hochberg and Bonferroni procedures, respectively, in steps 2 and 3 along with Guo et al. [17] procedure, under independence among genes and within genes

We summarize the results of the simulation studies in Fig. 1 for the independence case. The horizontal axis denotes the proportion of truly differentially expressed genes on the array and the vertical axis denotes the average mdFDR (left panel) and average power (right panel). As desired, all five procedures control the mdFDR on average at *α*=0.05. However, the proposed Dunnett based procedure has highest power compared to all other methods considered

**Fig. 2:**
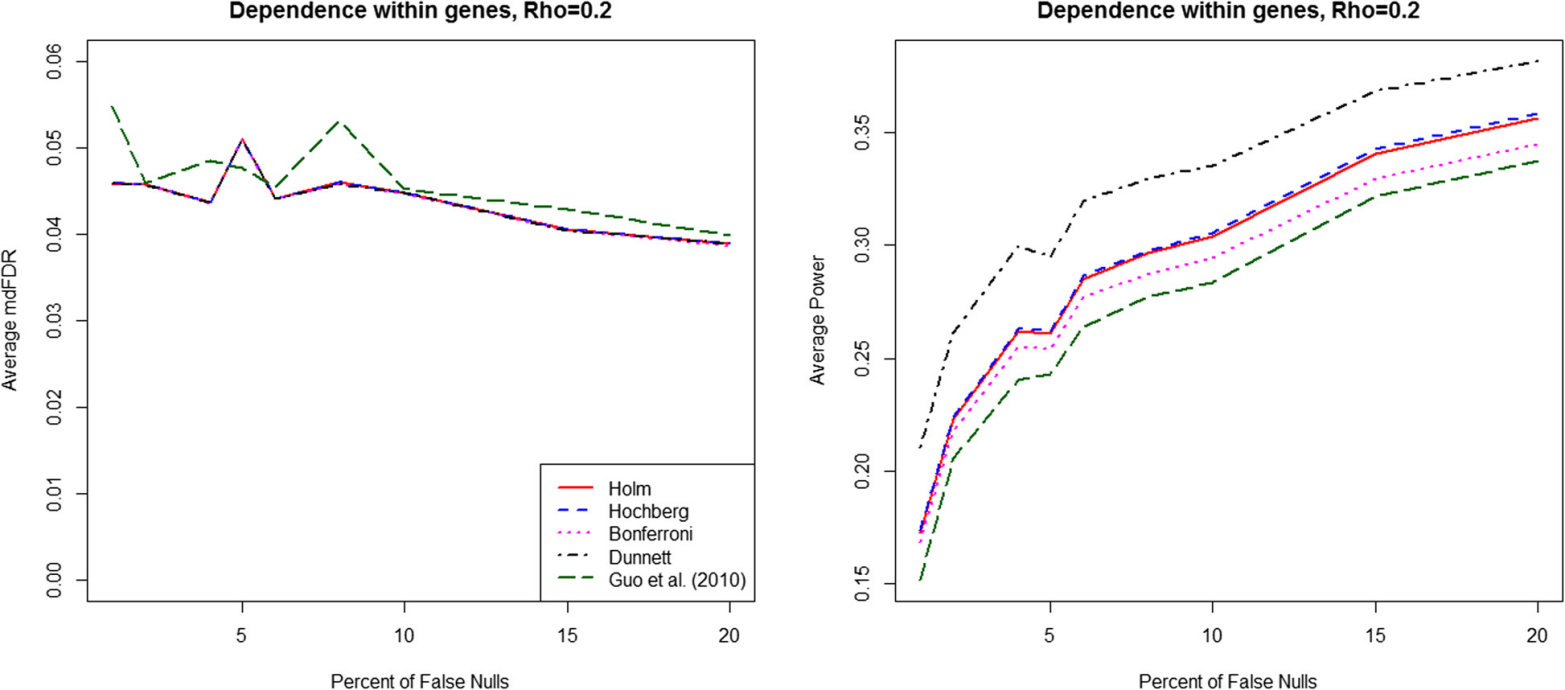
mdFDR (*left*), Average Power (*right*). mdFDR (*left*), Average Power (*right*) with the proposed methodology and three variants using Holm, Hochberg and Bonferroni procedures, respectively, in steps 2 and 3 along with Guo et al. [17] procedure, under dependence (*ρ*=0.2) within genes

**Fig. 3:**
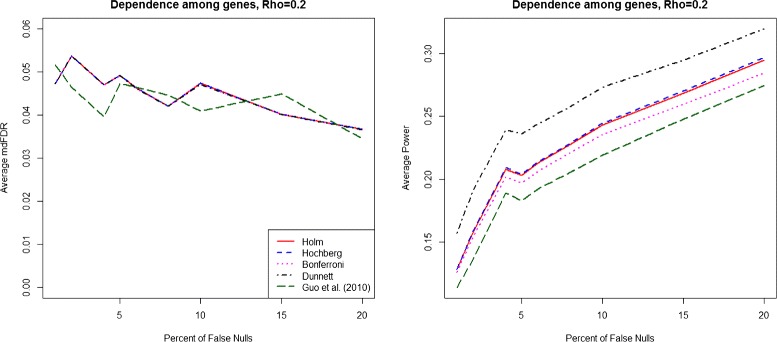
mdFDR (*left*), Average Power (*right*). mdFDR (*left*), Average Power (*right*) with the proposed methodology and three variants using Holm, Hochberg and Bonferroni procedures, respectively, in steps 2 and 3 along with Guo et al. [17] procedure, under dependence (*ρ*=0.2) among genes

We summarize the results for the case of dependence within genes across groups in Fig. 2, Additional file 2: Figure S2 and Additional file 3: Figure S3 and the results for the case of dependence among genes in Fig. 3, Additional file 4: Figure S4 and Additional file 5: Figure S5. The details of these dependence structures are given in the online Additional file 6: Section S4. Although all five procedures control the mdFDR on average at 0.05, the power of our proposed methodology is much higher compared to all four competing procedures under the different dependence structures considered.

### Analysis of gene expressions in uterine fibroids

Uterine fibroids are benign smooth muscle tumors occurring in the uterus of women with a very high prevalence rates in many populations. These tumors are considered to be hormonally mediated and therefore occur during the pre-menopausal years. According to some estimates, the cumulative incidence by age 50 of these tumors among Caucasian women exceeds 70 % and it is much higher among women of African American descent. The direct and indirect annual cost of fibroids in the US is as high as 34 billion dollars, yet these tumors are not well-studied perhaps because they are considered to be benign. The NIEHS conducted a large prospective study tracking the growth of fibroids in 72 pre-menopausal women over 12 months and discovered difference in growth patterns of fibroids according to age for white and black women [22]. A total of 52 tumor samples and 8 normal myometrium samples were collected from 12 women who opted for either hysterectomy or myomectomy. The RNA from these tissue samples were processed to obtain gene expression data using Affymetrix chips (Human Genome U133 plus 2.0). A total of 54675 probe sets were obtained. Using these gene expression data Davis et al. [29] studied the molecular characteristics of fibroids. Several important DEGs and canonical pathways were identified in Davis et al. [29], which were also experimentally validated using RtPCR.

**Fig. 4:**
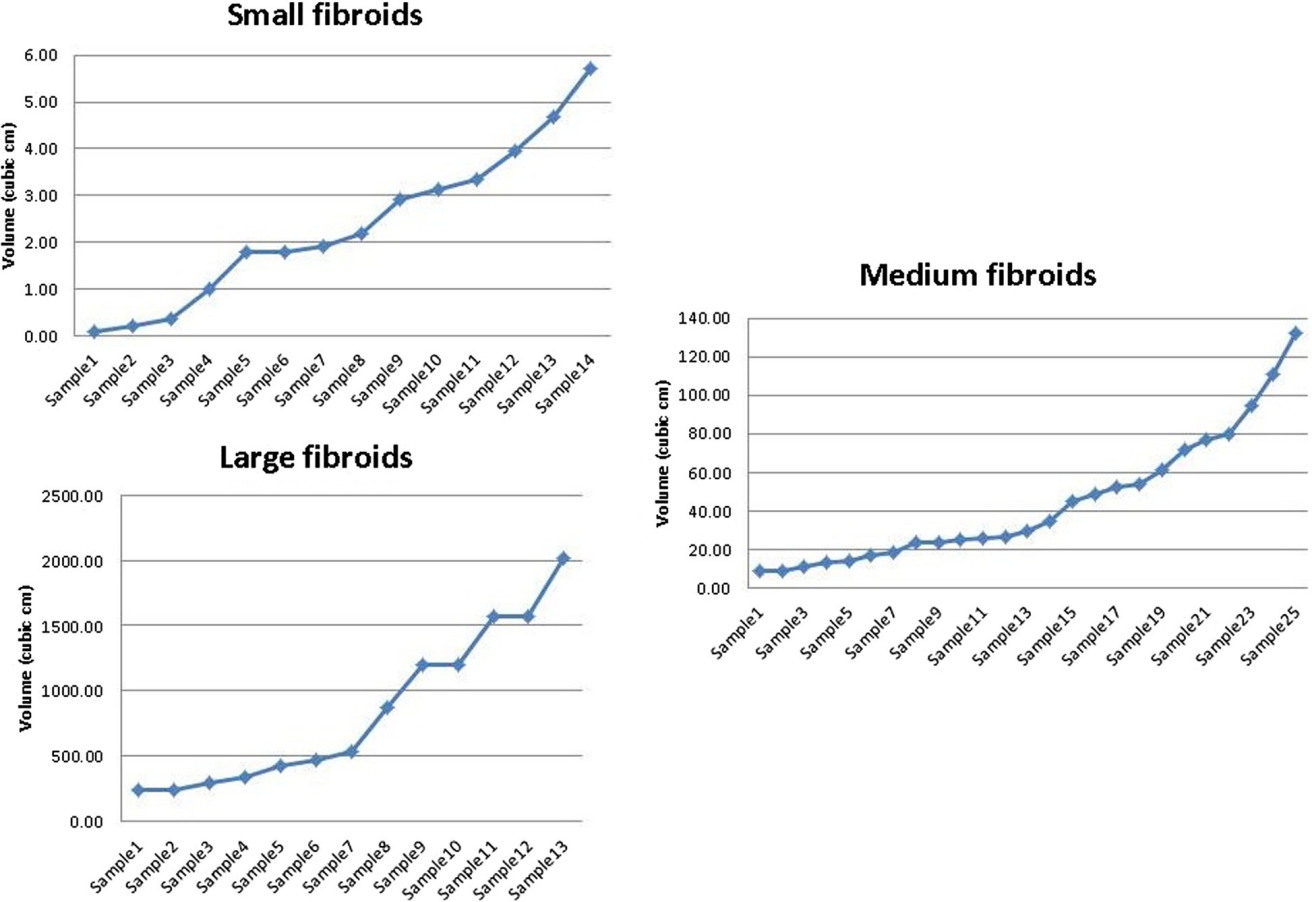
Volume of fibroids according to size category

In this paper we take the next step towards the understanding of molecular characteristics of fibroids by investigating the changes in gene expression according to tumor size. As done in the literature for other tumors such as breast cancer tumors and others [5–9], we are interested in identifying genes that are specific to size of uterine fibroids. We classified fibroids into three size groups according to their volume, small (14 samples, volume: 0.08–5.70 *cm*^3^), medium (25 samples, volume: 9.0–132.00 *cm*^3^) and large (13 samples, volume: 240-2016 *cm*^3^) and compared each size category with the normal myometrium, the reference group, using the proposed methodology. The cut points between size categories were chosen so that there is a natural separation between the size categories. The sorted volumes of the 52 tumors are provided in Fig. 4. Due to large differences in the volumes between large and small tumors, for clarity we provided volumes in three panels according to their size.

**Fig. 5:**
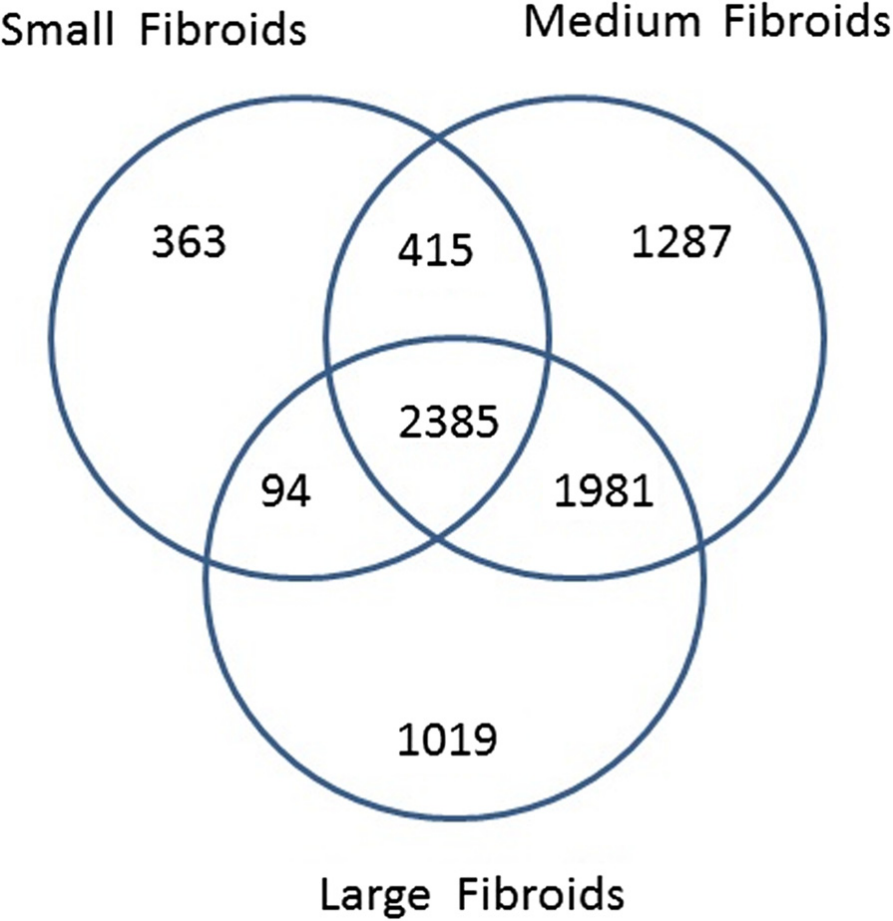
Venn diagram of differentially expressed genes by tumor size

**Fig. 6:**
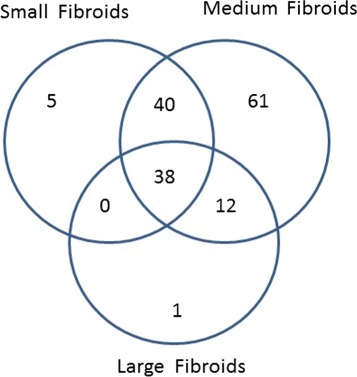
Venn diagram of enriched pathways by tumor size

We identified a total of 9553 probe sets to be differentially expressed in at least one pairwise comparison (relative to the normal myometrium) at mdFDR of 0.05. These 9553 probe sets map to 6286 genes. The Venn diagram of the DEGs by tumor size is in Fig. 5. Based on the 6286 genes, using the Ingenuity Pathway Analysis (IPA, 2000-2014 QIAGEN), we discovered a total 157 distinct enriched canonical pathways. The Venn diagram of the number of enriched canonical pathways by tumor size is provided in Fig. 6.

It is not surprising that a third of the DEGs and nearly 30 % of all enriched pathways are common to tumors of all sizes since tumor tissues are fundamentally different from the normal myometrium. However, we discover several DEGs and pathways that are uniquely enhanced according to the tumor size, suggesting that changes in molecular characteristics might be taking place as tumors grow. We highlight some important groups of genes and pathways discovered by our methodology and provide the complete list of all genes and pathways in the Additional files 7 and 8, respectively, for researchers to mine our data. The “Gene Symbol” and “Gene Name” columns in these tables are obtained from the website DAVID (http://david.abcc.ncifcrf.gov/).

*Growth factors, vascularization and related pathways*: The Netrin signaling pathway is well-known as a versatile pathway with multiple functions. One of its functions is to promote vascular networks and branching of blood vessels [30] and angiogenesis [31]. According to our analysis this pathway is uniquely enriched in small tumors only. The Interleukin-1 (IL-1) pathway is known to induce inflammatory response and the production of prostaglandins and expression of IL-2 which may play a critical role in the fibroid initiation and early development. For example, prostaglandins play a critical role in the promotion of growth factors involved in angiogenesis, such as VEGF, basic fibroblast growth factor (bFGF) and platelet-derived growth factor (PDGF) [32] and development of fibroid requires vascularization and blood supply. Thus Interleukin-1 (IL-1) pathway likely plays an important role during fibroid development. Interestingly, according to our analysis, this pathway is uniquely enriched in small size tumors but not in the medium or large tumors (Additional file 8). Furthermore, the fibroblast growth factors 8 and 20 (FGF8, FGF20) which belong to the Regulation of the Epithelial-Mesenchymal Transition Pathway and are well-known to be involved in vascularization and angiogenesis, are both uniquely down-regulated in small tumors and not differentially expressed in medium or large tumors. Our analysis further implies that the Epithelial-Mesenchymal Transition Pathway was enriched only in small and medium size tumors.

According to Ciarmela et al. [12], estrogen may promote fibroid growth through up-regulation of epidermal growth factor receptor (EGFR). However, we found EGFR to be down-regulated in fibroids and that too only in the medium size tumors. Similarly, the fibroblast growth factors (FGF) (acidic and basic) were differentially expressed only in the medium size tumors. The acidic FGF was up-regulated whereas the basic FGF was down regulated. These findings are consistent with Ciarmela et al. [12] (and references therein, e.g. Wolanska et al. [33]) in that they are expressed during tumor progression. Similarly, insulin like growth factor (IGF1) was only differentially expressed (up-regulated) in medium size tumors. Additionally, growth factor signaling pathways such as VEGF, PDGF, TGF *β* and EGF are uniquely enriched in medium size tumors and not in small or large tumors. While the above results suggest that some growth factors and related pathways are very specific to small and medium tumors, we discovered several growth factors to be differentially expressed in tumors of all sizes. These included, insulin like growth factor 2 (INS-IGF2), insulin like growth factor binding protein 5 (IGFBP5), and platelet derived growth factor C (PDGFC) which were up-regulated whereas insulin-like growth factor binding protein 6 (IGFBP6), connective tissue growth factor (CTGF), heparin-binding EGF-like growth factor (HBEGF), transforming growth factor beta receptor II (TGFBR2), fibroblast growth factors 12 and 13 (FGF12, FGF13) were down-regulated. Similarly, growth factor signaling pathways such as the human growth factor (HGF) and IGF-1 were enriched in tumors of all sizes. Thus it appears that the differential expression of these genes and the enrichment of the above pathways is necessary for tumor onset and progression.

*Estrogen and related genes*: Fibroids are hormonally mediated and it is also well documented in the literature that accordingly estrogen and progesterone receptors and prostaglandins promote proliferation of fibroids (see [29, 34]). Not surprisingly, we found the estrogen receptor ESR-1 to be up-regulated in tumors of all sizes. This result was confirmed using the RtPCR data (see Additional file 9). Our RtPCR data further indicates that the expression of ESR-1 in small tumors is almost 38 % higher than in large tumors, suggesting that ESR-1 has a large effect on small tumors but is also needed for the continued growth of the tumor. Interestingly, the progesterone receptor (PGR) was up-regulated in only medium size tumors and not differentially expressed in small or large tumors, a result that is also confirmed by the RtPCR (Additional file 9). This suggests that perhaps PGR may not be involved in tumor initiation (i.e. small tumors) but is only involved in growth of the tumor. However, its function ends once the tumor becomes large enough. Most prostaglandins were generally down-regulated in tumors of all sizes. For example, prostaglandin E receptor 3 (PTGER3), prostaglandin F receptor (PTGFR) and prostaglandin-endoperoxide synthase 2 (prostaglandin G/H synthase and cyclooxygenase) (PTGS2) are down-regulated in tumor of all sizes. The result of PTGS2 was also confirmed using RtPCR (see Additional file 9). However, some prostaglandins were differentially expressed according to the size of the tumor. For example, prostaglandin-endoperoxide synthase 1 (prostaglandin G/H synthase and cyclooxygenase) (PTGS1) was differentially expressed only in the medium sized tumors where it was down-regulated and prostaglandin E synthase 2 (PTGES2) was down-regulated in large tumors only but prostaglandin E receptor 4 (PTGER4) was down-regulated in both medium and large tumors.

Similar to estrogen and progesterone receptors, the *α* and *γ* isoforms of peroxisome proliferator-activated (PPAR) receptors have been associated with the regulation of proliferation of uterine fibroids [35, 36]. In our data both these isoforms are down-regulated in all tumor sizes compared to the normal myometrium. We also discovered the related retinoid X receptor gamma to be down-regulated in the medium size tumors but was not significant in small or large tumors.

*Collagens*: There is a vast amount of literature implicating collagens to smooth muscle tumors such as the fibroids (see [29], and references therein). Consequently, it is not surprising that several collagens (COL1A1, COL1A2, COL3A1, COL4A1-COL4A4, COL5A2, COL6A3, COL7A1, COL9A2, COL21A1, COL22A1, COL27A1) and extra cellular matrix proteins are differentially expressed in tumors of all sizes. Apart from COL4A3, COL4A4 and COL21A1, which were down-regulated in all tumor size groups, the remaining 11 collagens were up-regulated in tumor samples. We investigated a subset of these collagens using RtPCR which confirmed the above findings for those genes (see Additional file 9). There were other collagens that were differentially expressed depending upon the size of the tumor. For example, COL2A1, COL4A6 and COL5A1 were not differentially expressed in small tumors but were significantly up-regulated in medium and large tumors. Collagen COL23A1 was significantly up-regulated in small and medium tumors but not in the large fibroids. Interestingly, some of the collagens were differentially expressed in only one of the size categories. For example, COL4A3BP, COL6A1, COL9A3, COL10A1 and COL11A1 were differentially expressed only in the large tumors. Again, except for COL4A3BP, the remaining 4 collagens were up-regulated in the tumor samples. Interestingly, COL5A3 was only differentially expressed (down-regulated) in small tumors.

*Other genes*: Leptin receptor is well-known to be negatively associated with the obesity and obesity is a potential risk factor for fibroids. Interestingly, we discover leptin receptor (LEPR) to be significantly down-reglated in all tumor sizes. This finding is also confirmed by the RtPCR data reported in Additional file 9. As noted earlier, Diaz at al. [5] demonstrated that *β*4 integrin had an increased expression in larger breast tumors and in higher tumor grades. In our fibroid data, however, we notice a down-regulation in *β*4 integrin in medium and large tumors and was not differentially expressed in small tumors.

### Comparison of various procedures with Dunnett’s test based methodology

**Fig. 7:**
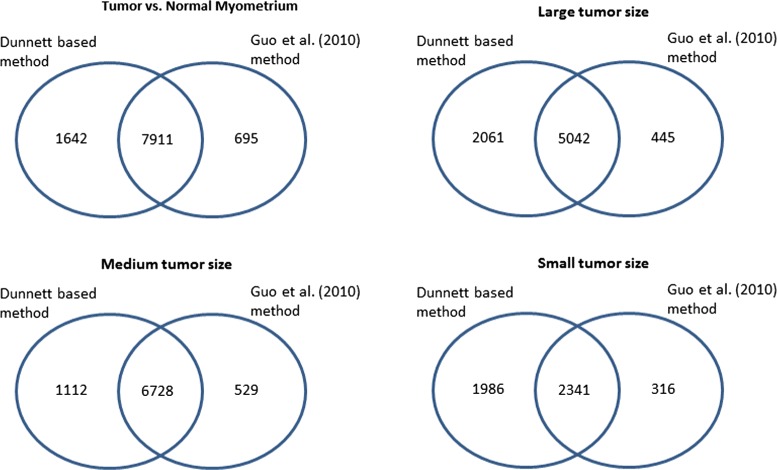
Venn diagram of identified probe sets by the Dunnett based methodology and Guo et al. [17] method. The Venn diagrams compare the two procedures in terms of the probe sets identified in at least one tumor size, Large tumor size (irrespective of being identified in medium or small tumor sizes), Medium tumor size (irrespective of being identified in Large or Small tumor sizes) and Small tumor size (irrespective of being identified in Large or Medium tumor sizes)

**Fig. 8:**
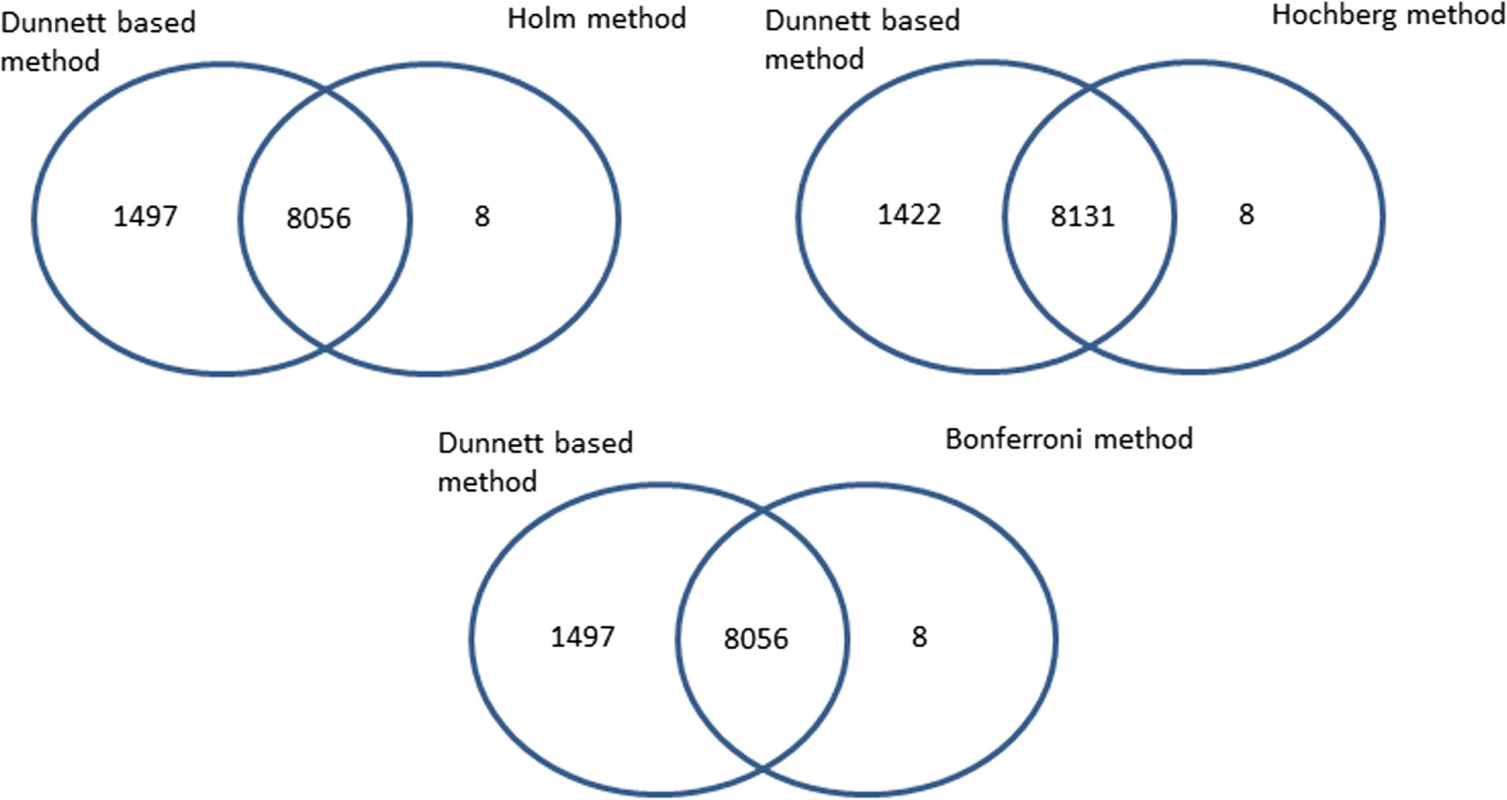
Venn diagram of identified probe sets by the Dunnett based methodology and other methods discussed in numerical studies. The other methods considered in the numerical simulation are Dunnett screening and Holm procedure (Holm), Dunnett screening and Hochberg procedure (Hochberg) and Dunnett screening and Bonferroni procedure (Bonferroni)

We analyzed the NIEHS FGS data using various methods considered in this paper at mdFDR level of 0.05. We compared the methods in terms of number of probe sets that were differentially expressed (relative to normal myometrium) in; (a) all tumors, (b) small tumors, (c) medium tumors, and (d) large tumors. Results are summarized using Venn diagrams. In Fig. 7 we compare our Dunnett test based methodology with Guo et al. [17] procedure. Not only did the Dunnett based methodology identified many of the probe sets identified by the Guo et al. [17] procedure, i.e. shared several probe sets (overall = 7911, small = 5042, medium = 6728, large = 2341), it identified several more that were not identified by Guo et al. [17] procedure (overall = 1642, small = 2061, medium = 1112, large = 1986). On the contrary, there were very few probe sets that were uniquely identified by Guo et al. [17] procedure (overall = 695, small = 445, medium = 529, large = 316). We see similar results when we compare Dunnett screening and Holm procedure (Holm), Dunnett screening and Hochberg procedure (Hochberg) and Dunnett screening and Bonferroni procedure (Bonferroni). The Venn diagram in Fig. 8 compares the number of probe sets identified by Dunnett based method to each of the other methods. In each case the Dunnett based method tends to uniquely identify many probe sets than each of the other methods. The total number of probe sets identified by the various methods are summarized in Table 1.

**Table 1:** Probe sets identified by our suggested Dunnett based method, Guo et al. [17] method and other possible methods

	Dunnett based	Guo et al.	Holm	Hochberg	Bonferroni
At least one tumor size	9553	8606	8064	8139	8064
Large tumors only	1180	922	503	483	697
Medium tumors only	1548	2303	1557	1489	1976
Small tumors only	427	349	175	162	224
Large and Medium tumors only	2498	2724	2168	2189	2712
Large and Small tumors only	106	78	40	45	77
Medium and Small tumors only	475	467	331	339	475
All tumor sizes	3319	1763	3290	3432	1903

## Conclusion

This paper offers a general statistical methodology for conducting pairwise comparisons in high dimensional data that is broadly applicable to a wide range of genomic data including gene expression microarray data, CpG methylation data, RNA-seq data and others and proposes a specific testing methodology for the analysis of FGS gene expression data. The proposed methodology not only controls the false discoveries made when making several pairwise comparisons on the basis of high dimensional data, but it controls for directional errors, i.e. falsely declaring a gene to be up-regulated (down-regulated) when it is not. Thus it controls the mixed directional false discovery rate (mdFDR). The framework we proposed here is general enough that, depending upon the application, a researcher can use different testing procedures within our framework. In all situations the procedure strongly controls mdFDR. For the application under consideration in this paper, the experimental design calls for the comparison of several experimental groups against a particular group. For such designs, in the classical multiple comparison problem, the Dunnett’ test is known to be powerful. So, it is not surprising that our Dunnett’s test based procedure is most powerful among all the procedures we considered in this paper. Our methodology based on Dunnett procedure works best here in terms of power because (a) the Dunnett’s *p*-values are based on the joint distribution of the test statistics and (b) our procedure uses the maximum order statistic to find the screening *p*-value.

Using our methodology we gain deeper insights into molecular characteristics of uterine fibroids according to the tumor size. We have identified several differentially expressed genes and pathways that are specifically enriched according to the tumor size. While researchers and clinicians who study fibroids are well aware of many of the genes and pathways described in this paper, we have provided a characterization of these genes and pathways according to the tumor size. Our data can be further mined to gain deeper insights regarding fibroids. In view of the recent publication [29] we acknowledge that our results are potentially confounded by race. Since the sample size by race is very small, we are unable to separate out the race effect. Despite that potential weakness, we think that our findings provoke researchers to explore further research along these lines.

## Additional files

Additional file 1
**Figure S1.** A graphical display of various hypotheses of interest. (TIFF 149 kb)

Additional file 2
**Figure S2.** mdFDR (left), Average Power (right) with the proposed methodology and three variants using Holm, Hochberg and Bonferroni procedures, respectively, in steps 2 and 3 along with Guo et al. [17] procedure, under dependence (*ρ*=0.5) within genes. (TIFF 37 kb)

Additional file 3
**Figure S3.** mdFDR (left), Average Power (right) with the proposed methodology and three variants using Holm, Hochberg and Bonferroni procedures, respectively, in steps 2 and 3 along with Guo et al. [17] procedure, under dependence (*ρ*=0.8) within genes. (TIFF 36 kb)

Additional file 4
**Figure S4.** mdFDR (left), Average Power (right) with the proposed methodology and three variants using Holm, Hochberg and Bonferroni procedures, respectively, in steps 2 and 3 along with Guo et al. [17] procedure, under dependence (*ρ*=0.5) among genes. (TIFF 36 kb)

Additional file 5
**Figure S5.** mdFDR (left), Average Power (right) with the proposed methodology and three variants using Holm, Hochberg and Bonferroni procedures, respectively, in steps 2 and 3 along with Guo et al. [17] procedure, under dependence (*ρ*=0.8) among genes. (TIFF 36 kb)

Additional file 6
**Supplementary Materials.** **Section S1.** The mathematical notations and definitions used in the paper are described in this section. The hypotheses tested in different steps of the testing procedure are formulated and interpreted. **Section S2.** The mathematical proof of the theorem that states that the general testing algorithm controls mdFDR. **Section S3.** The details of the statistical methodology developed for the testing and analysis of the FGS microarray data. **Section S4.** The supplementary results for the simulation study performed to evaluate the power of the developed statistical methodology compared to several other competing methods. (PDF 270 kb)

Additional file 7
**Differentially expressed probe sets in different sizes of tumors compared to normal myometrium.** Significant probe sets are grouped and color coded according to different sizes of fibroids. The last column shows the BH-adjusted p-value for step 1 of the methodology. The columns under "Dunnett Adjusted P-values" show the p-values for step 2 of the methodology. (XLSX 1863 kb)

Additional file 8
**Pathways identified in different sizes of tumors compared to normal myometrium.** In the table, the enriched pathways are grouped and color coded according to different sizes of fibroids. The last column shows the negative of the logarithm of the BH adjusted p-value, computed by IPA. (XLSX 15 kb)

Additional file 9
**RtPCR data.** This table shows the RtPCR data for a subset of the genes that are identified in our analysis. (XLSX 11 kb)
