# Supplementary figures and images for: A multiple testing procedure for multi-dimensional pairwise comparisons with application to gene expression studies
Source: BMC Bioinformatics. 2016 Feb 25;17:104. doi: 10.1186/s12859-016-0937-5 (PMC4768411; doi:10.1186/s12859-016-0937-5)

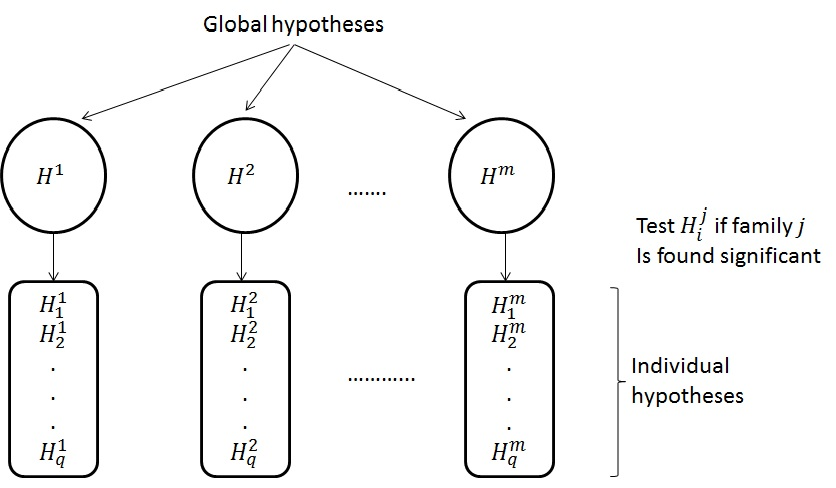

Supplement: Additional file 1 — Figure S1. A graphical display of various hypotheses of interest. (TIFF 149 kb) [file 12859_2016_937_MOESM1_ESM.tiff]

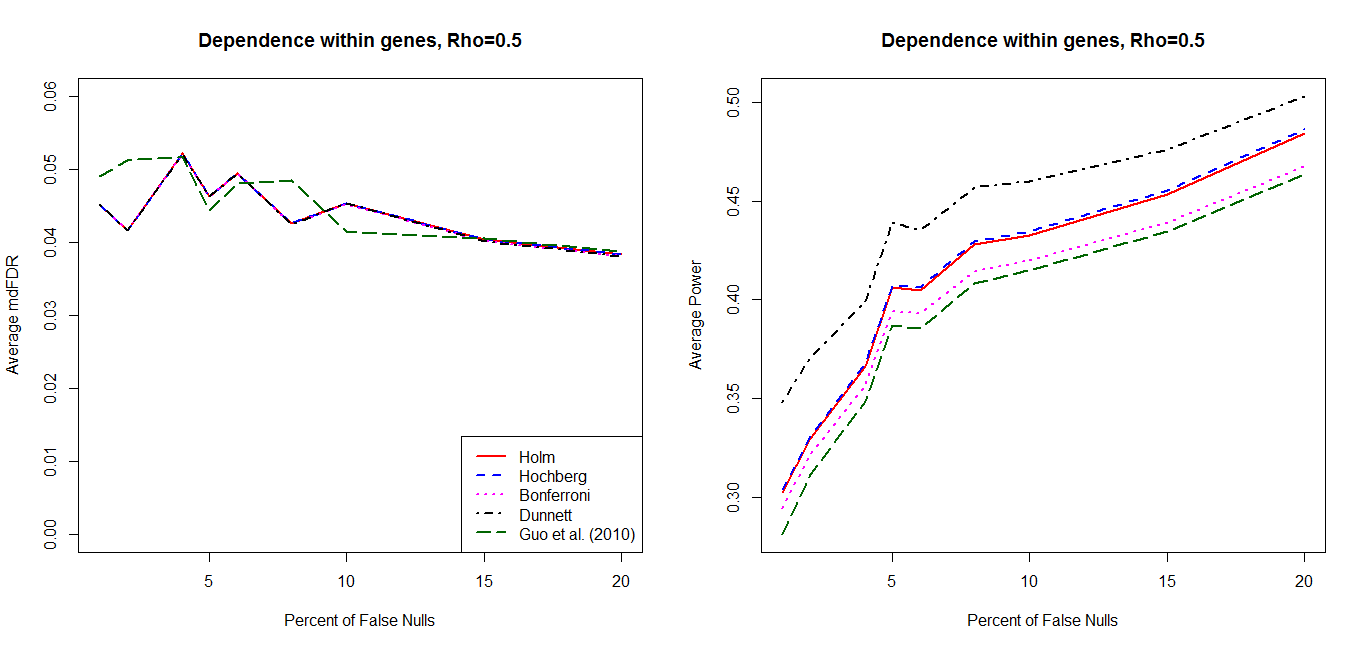

Supplement: Additional file 2 — Figure S2. mdFDR (left), Average Power (right) with the proposed methodology and three variants using Holm, Hochberg and Bonferroni procedures, respectively, in steps 2 and 3 along with Guo et al. [17] procedure, under dependence (ρ=0.5) within genes. (TIFF 37 kb) [file 12859_2016_937_MOESM2_ESM.tiff]

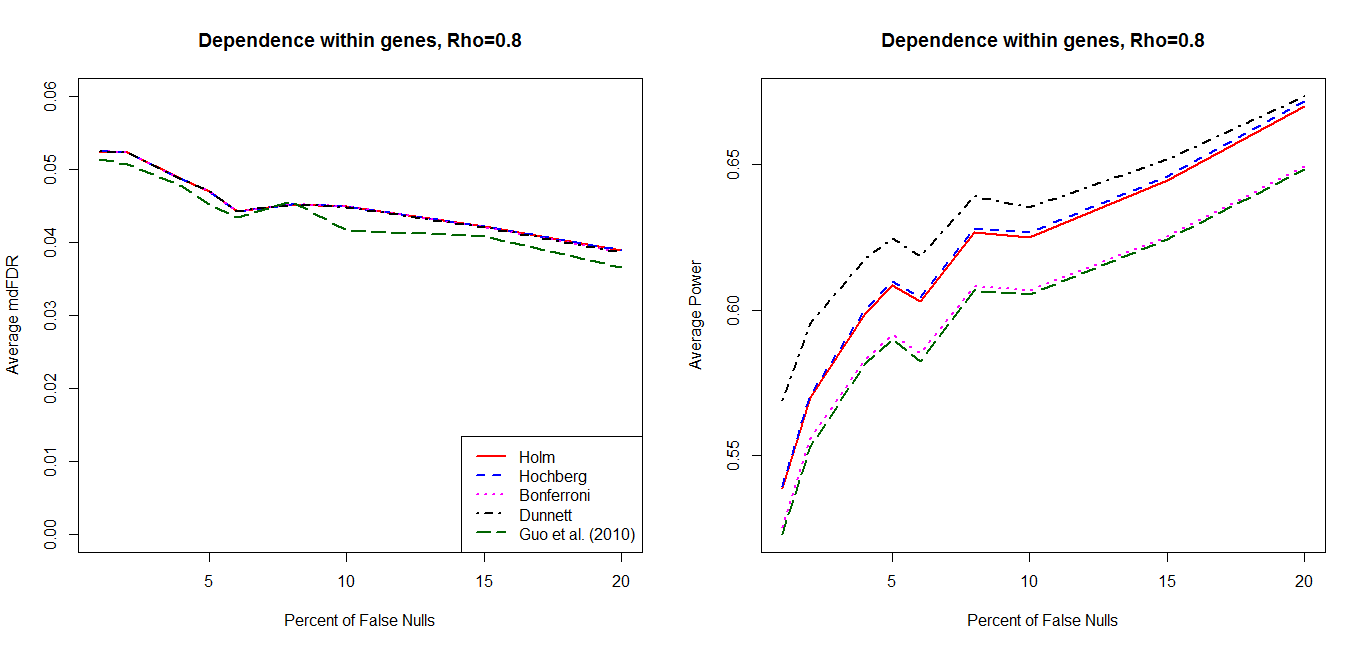

Supplement: Additional file 3 — Figure S3. mdFDR (left), Average Power (right) with the proposed methodology and three variants using Holm, Hochberg and Bonferroni procedures, respectively, in steps 2 and 3 along with Guo et al. [17] procedure, under dependence (ρ=0.8) within genes. (TIFF 36 kb) [file 12859_2016_937_MOESM3_ESM.tiff]

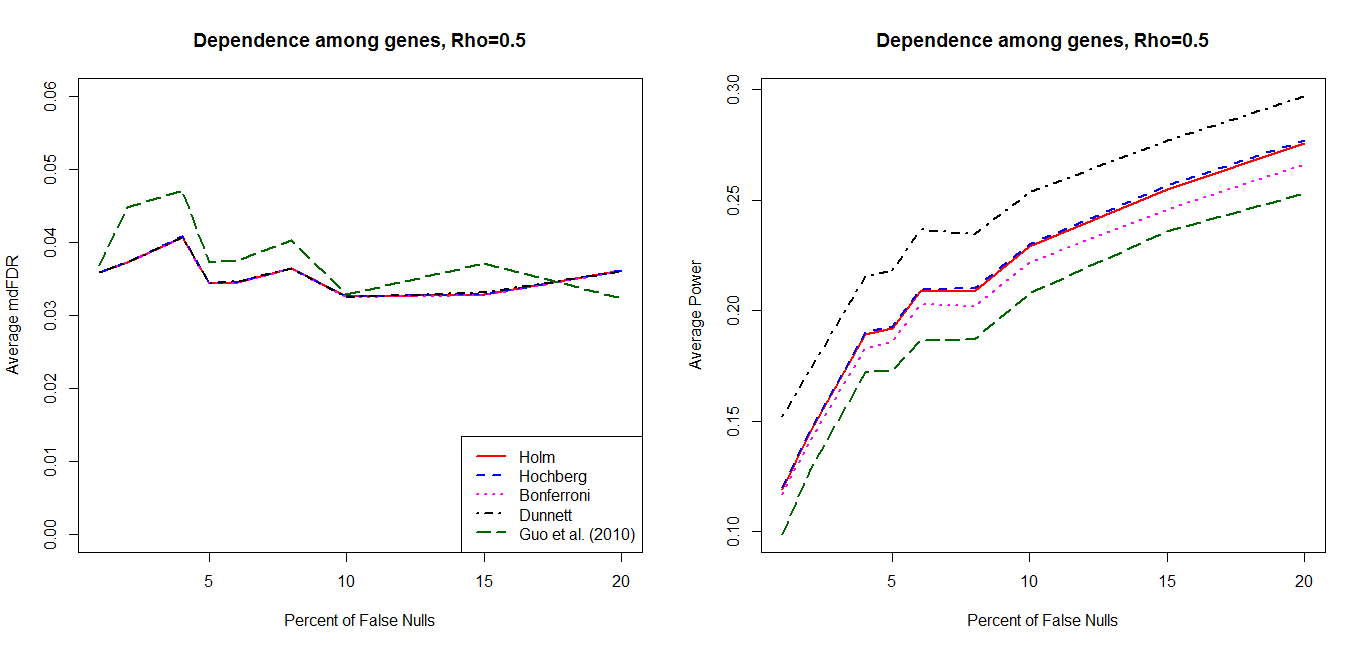

Supplement: Additional file 4 — Figure S4. mdFDR (left), Average Power (right) with the proposed methodology and three variants using Holm, Hochberg and Bonferroni procedures, respectively, in steps 2 and 3 along with Guo et al. [17] procedure, under dependence (ρ=0.5) among genes. (TIFF 36 kb) [file 12859_2016_937_MOESM4_ESM.tiff]

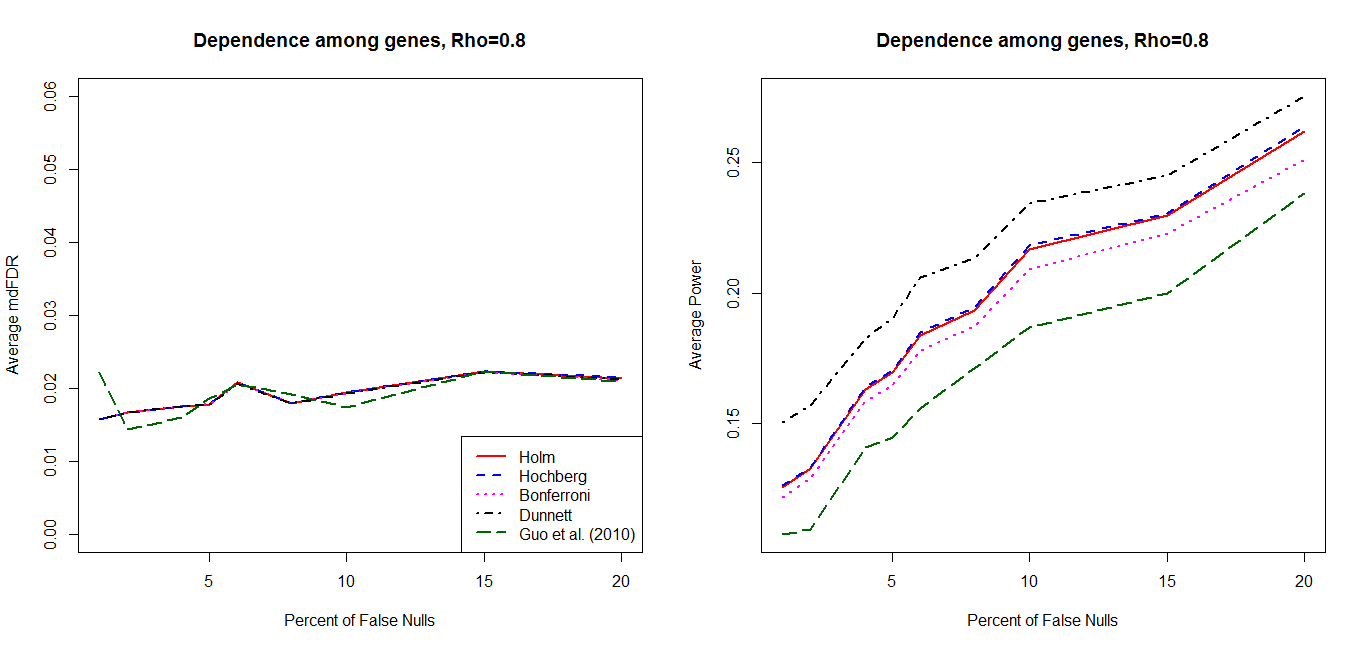

Supplement: Additional file 5 — Figure S5. mdFDR (left), Average Power (right) with the proposed methodology and three variants using Holm, Hochberg and Bonferroni procedures, respectively, in steps 2 and 3 along with Guo et al. [17] procedure, under dependence (ρ=0.8) among genes. (TIFF 36 kb) [file 12859_2016_937_MOESM5_ESM.tiff]
