# Supplementary material for: A multiple testing procedure for multi-dimensional pairwise comparisons with application to gene expression studies
Source: BMC Bioinformatics. 2016 Feb 25;17:104. doi: 10.1186/s12859-016-0937-5 (PMC4768411; doi:10.1186/s12859-016-0937-5)
Supplement: Additional file 6 — Supplementary Materials. Section S1. The mathematical notations and definitions used in the paper are described in this section. The hypotheses tested in different steps of the testing procedure are formulated and interpreted. Section S2. The mathematical proof of the theorem that states that the general testing algorithm controls mdFDR. Section S3. The details of the statistical methodology developed for the testing and analysis of the FGS microarray data. Section S4. The supplementary results for the simulation study performed to evaluate the power of the developed statistical methodology compared to several other competing methods. (PDF 270 kb) [file 12859_2016_937_MOESM6_ESM.pdf]

Supplementary Materials: A multiple testing procedure for multi-dimensional pairwise comparisons with application to gene expression studies

Anjana Grandhi, Wenge Guo, Shyamal D. Peddada

S1 Notations and Definitions

Let "m" denote the number of genes in the data that has gene expressions for each gene on "p" categories (tumor sizes and normal tissue). Let μ_{ij} denote the mean response corresponding to the i-th category in j-th gene, $i = 1, ..., p$, $j = 1, ..., m$. The problem of biological interest we discuss in the context of uterine fibroid data is to detect genes that are differentially expressed in a tumor size category compared to normal sample. Thus, if the last category, "p", corresponds to normal sample, we need to test the pairwise differences $\theta_{ij} = \mu_{ij} - \mu_{pj}$, $i = 1, 2, ..., q$ and $j = 1, 2, ..., m$, are equal to zero, where, $q = p - 1$. For each gene j, the pairwise null and alternative hypotheses are,

$$H_{0i}^{j} : \theta_{ij} = 0 \text{ against } H_{1i}^{j} : \theta_{ij} \neq 0, \quad i = 1, \dots, q. \tag{S1}$$

For each gene j, we have a vector of parameters $\boldsymbol{\theta}_j = (\theta_{1j}, \theta_{2j}, ..., \theta_{qj})$. We first need to find out the genes that are differentially expressed in at least one tumor sample compared to normal samples. Thus, we define the null and alternative "screening hypotheses" to test the significance of each gene as,

$$H_{0screen}^{j} : \boldsymbol{\theta}_j = \mathbf{0} \text{ against } H_{1screen}^{j} : \boldsymbol{\theta}_j \neq \mathbf{0}, \quad j = 1, \dots, m, \tag{S2}$$

for testing whether all parameters θ_{ij}, $i = 1, ..., q$ are simultaneously 0 or not, equivalently, whether all $\mu_{ij}, i = 1, ..., p$ are equal or not. These hypotheses give rise to m families of hypotheses corresponding to m genes with each family having a screening hypothesis and"q" pairwise hypotheses. Figure S1 shows a simple graphical representation of the structure of hypotheses in our formulation.

Let x_{ij}^{k} denote the kth observed gene expressions of the jth gene in i^{th} group, $k = 1, \dots, n_i$ with n_i being the sample size for i^{th} group, $j = 1, \dots, m$, $i = 1, \dots, p$. Let T_{ij} and P_{ij}, $i = 1, \dots, q$, $j = 1, \dots, m$, denote the test statistics and the p-values respectively for testing H_{0i}^{j}. The test statistics for testing the screening hypotheses $H_{0screen}^{j}$ are obtained as a function of $T_{ij}, i = 1, \dots, q$, for instance, the highest order statistic of $T_{ij}, i = 1, \dots, q$. We denote the p-values for testing screening hypotheses as P_{screen}^{j}. For each family j we denote a vector of p-values, $\mathbf{P_j} = (P_{1j}, P_{2j}, ..., P_{qj})$ based on the test statistics $\mathbf{T_j} = (T_{1j}, T_{2j}, ..., T_{qj})$, for testing the pairwise hypotheses in (S1). If H_{0i}^{j} is rejected we conclude on direction, i.e., declare $\theta_{ij} > 0$ if $T_{ij} > 0$ or declare $\theta_{ij} < 0$ if $T_{ij} < 0$.

Given the screening p-values P_{screen}^{j}, for every $j = 1, 2, ..., m$, to carry out the simultaneous testing of the screening hypotheses in (S2), we use the BH-procedure [1], that controls the FDR at a given level α. This is a step-up procedure as follows: given the ordered screening p-values $P_{screen}(1) \leq P_{screen}(2) \leq \cdots \leq P_{screen}(m)$ and the corresponding screening null hypotheses $H_{0screen}(1), H_{0screen}(2), ..., H_{0screen}(m)$,

find, $R = \max\{1 \le j \le m : P_{screen}(j) \le j\alpha/m\}$ and reject $H_{0screen}(1), \ldots, H_{0screen}(R)$, provided the maximum exists, otherwise, accept all the screening hypotheses.

When an $H^j_{0screen} : \boldsymbol{\theta}_j = \mathbf{0}$ is rejected, further decisions are made on the pairwise hypotheses in (S1) and on rejection, directional decisions are made on the signs of the component θ_{ij}. A Type I error might occur due to wrongly rejecting $H^j_{0screen}$ or correctly rejecting $H^j_{0screen}$ but wrongly rejecting H^j_{0i} for some $i = 1, \ldots, q$. A Type II error might occur due to failing to reject a false null hypothesis $H^j_{0screen}$ or correctly rejecting a false $H^j_{0screen}$ but failing to reject a false null pairwise hypothesis H^j_{0i} for some $i = 1, \ldots, q$. A directional error (Type III error) might occur due to correctly rejecting $H^j_{0screen}$ but wrong assignment of the sign of θ_{ij} while correctly rejecting $H^j_{0i} : \theta_{ij} = 0$. The general practice in any multiple testing problem is to find a procedure that controls the Type I errors and minimizes the Type II errors. Here, we need to control Type I as well as Type III errors. A practical way of doing that would be to use an error rate combining both Type I and Type III errors in the FDR framework and make sure that it is controlled.

An error rate that combines Type I errors and Type III errors in FWER setup is mdFWER [2, 3], which is the probability of making at least one Type I error or Directional error. Heller et al. [4] used the Overall False Discovery Rate (OFDR), which is the expected proportion of falsely discovered gene sets out of all discovered gene sets, as an appropriate error measure to control, in their two-stage procedure for identifying differentially expressed genes and gene sets. The concept of OFDR was introduced by Benjamini and Heller [5] in the context of testing partial conjunction hypotheses. Inspired by Heller et al. [4] and Shaffer [2], Guo et al. [6] define the mixed directional False Discovery Rate (mdFDR) defined below.

Let $V(j)$ denote the indicator function of at least one Type I error or Directional Error committed while testing family j and the pairwise hypotheses in it, i.e., $V(j)$ is 1 if either $H^j_{0screen}$ is falsely rejected or $H^j_{0screen}$ is correctly rejected but at least one Type I error or Directional error occurs while testing pairwise hypotheses in the family j; $V(j)$ is 0 otherwise. Let, R denote the number of screening hypotheses rejected by a multiple testing procedure, that is, $R = \max\{1 \le j \le m : P_{screen}(j) \le j\alpha/m\}$. Then, mdFDR is formally defined as follows.

Definition 1: *mdFDR - mixed directional False Discovery Rate.* The expected proportion of Type I and Directional errors among all discovered families,

$$mdFDR = E\left[\frac{\sum_{j=1}^{m} V(j)}{\max(R,1)}\right]. \tag{S3}$$

S2 Proof of Theorem 1

Proof Let, I_0 denote the set of true null screening hypotheses $H^j_{0screen}$ and I_1 denote set of false $H^j_{0screen}$ with $|I_0| = m_0$ and $|I_1| = m_1$, $m_0 + m_1 = m$. From definition of mdFDR,

$$mdFDR = E(Q) = E\left[\frac{\sum_{j=1}^{m} V(j)}{\max(R,1)}\right]. \tag{S4}$$

Let, $P_{screen}(1) \le \cdots \le P_{screen}(m)$, denote the ordered screening p-values. In the event that $R = r$, $P_{screen}(k) \le r\alpha/m$ for $k = 1, 2, ..., r$ and $P_{screen}(k) > (r+1)\alpha/m$

for $k = r+1, ..., m$. Consequently, we have r number of P^j_{screen}'s that are $\leq r\alpha/m$. Then, (S4) can be written as,

$$\begin{aligned}
&E(Q)\\
&= \sum_{r=1}^{m} \frac{1}{r} \left[\sum_{j=1}^{m} Pr(V(j) = 1, R = r) \right]\\
&= \sum_{r=1}^{m} \sum_{j \in I_0} \frac{1}{r} Pr\left(P^j_{screen} \leq \frac{r\alpha}{m},\ R^{(-j)} = r-1 \right)\\
&\quad + \sum_{r=1}^{m} \sum_{j \in I_1} \frac{1}{r} Pr\left(P^j_{screen} \leq \frac{r\alpha}{m}, \text{Type I or}\right.\\
&\qquad\qquad \left. \text{Type III error at j}, R^{(-j)} = r-1 \right). \qquad \text{(S5)}
\end{aligned}$$

where, $R^{(-j)}$ denotes the number of screening hypotheses rejected from the set of $(m-1)$ screening hypotheses $\{H^1_{0screen}, H^2_{0screen}, ..., H^{j-1}_{0screen}, H^{j+1}_{0screen}, ..., H^m_{0screen}\}$ by using the step-up procedure with the critical values $(i+1)\alpha/m, i = 1, \ldots, m-1$.

First consider the second term in (S5). By the assumption of independence of p-value vectors we can write it as follows:

$$\begin{aligned}
&\sum_{r=1}^{m} \sum_{j \in I_1} \frac{1}{r} Pr\left(P^j_{screen} \leq \frac{r\alpha}{m}, \text{Type I or Type III error at j} \right)\\
&\qquad \cdot Pr\left(R^{(-j)} = r-1 \right)\\
&\leq \sum_{r=1}^{m} \sum_{j \in I_1} \frac{1}{r} \frac{r\alpha}{m} Pr\left(R^{(-j)} = r-1 \right) \qquad \text{(S6)}\\
&= \frac{m_1\alpha}{m}. \qquad \text{(S7)}
\end{aligned}$$

The inequality in (S6) follows as we use an mdFWER controlling procedure at level $(r\alpha/m)$ for each significant family and as $j \in I_1$, the probability of making at least one Type I error or directional error in family j is $\leq r\alpha/m$. Summing over all values of r, the equality in (S7) follows by noting that $\sum_{r=1}^{m} Pr\Big(R^{(-j)} = r-1\Big) = 1$.

Next consider the first term in (S5). By the assumption of independence of p-value vectors we can write it as follows:

$$\begin{aligned}
&\sum_{r=1}^{m} \sum_{j \in I_0} \frac{1}{r} Pr\left(P^j_{screen} \leq \frac{r\alpha}{m} \right)\\
&\qquad \cdot Pr\left(R^{(-j)} = r-1 \right)\\
&\leq \sum_{r=1}^{m} \sum_{j \in I_0} \frac{1}{r} \frac{r\alpha}{m} Pr\left(R^{(-j)} = r-1 \right) \qquad \text{(S8)}\\
&= \frac{m_0\alpha}{m}. \qquad \text{(S9)}
\end{aligned}$$

The inequality in (S8) follows due to the fact that the true null p-values are stochastically larger than or equal to $U(0,1)$. Summing over all values of r, the equality

in (S9) follows by noting that $\sum_{r=1}^{m} Pr\left(R^{(-j)} = r-1\right) = 1$. The result follows by combining (S7) and (S9). ■

In the theorem we only assume that the p-value vectors are independent and we do not discuss about the component pairwise p-values. This implies that the p-values within a gene across tumor samples may have any dependence structure. The mdFWER controlling procedure used will tell us under what kind of dependence structures of p-values within genes is the procedure valid. For example, if we use Holm's mdFWER controlling procedure, which is proved to control the mdFWER when the p-values are independent, then this theorem is valid under the additional assumption that the pairwise p-values $P_{ij}, i = 1, 2, \ldots, q$ of a vector $\mathbf{P_j}$ are independent.

The generality of this algorithm makes it a flexible procedure to apply to several practical situations where multidimensional directional decisions are required to make. Although, in the paper, we discuss testing of differential gene expression in each tumor size against the normal sample for each gene, this procedure can be applied to any type of pairwise comparison desired to be tested for each gene. For example, if it is of interest to group genes by the inequalities among the mean responses, we would want to detect the pattern of mean responses in the p categories, known as directional pattern, and see how the mean responses vary across the categories. Some common inequalities are $\mu_{1j} \leq \mu_{2j} \leq \cdots \leq \mu_{pj}$ (monotone pattern), $\mu_{1j} \leq \cdots \leq \mu_{ij} \geq \mu_{(i+1)j} \geq \cdots \mu_{pj}$ (umbrella pattern with peak μ_{ij}). To test for the pattern we need to test the differences of mean response of the categories, $\theta_{ij} = \mu_{i+1j} - \mu_{ij}$, $i = 1, 2, ..., q$ and $j = 1, 2, ..., m$ and $q = p - 1$. If the problem of interest is testing all pairwise differences of the p categories, possibly unordered, then $q = p(p-1)/2$. Based on the question we want to answer from a data, appropriate methodology can be developed from this general procedure.

S3 Details of Statistical Methodology for FGS Gene Expression Data

Dunnett P_{screen} for Step 1: The scenario is of comparing multiple groups with a common control group and the standard method used in this situation is the Dunnett test [7]. Dunnett test is a powerful method that is designed specifically for this kind of comparison. The test assumes that the underlying distributions of the data from the different groups have same variance and the test statistics are obtained by using a pooled estimate of the variance. This assumption is valid for the Uterine fibroid data as the gene expressions are normalized to have similar means and variances for comparability. The test statistic for testing (S1) is given by,

$$T_{ij}^{Dunn} = \frac{\bar{x}_{ij} - \bar{x}_{pj}}{s_j\sqrt{\frac{1}{n_i} + \frac{1}{n_p}}}, \tag{S10}$$

where, $\bar{x}_{ij} = (1/n_i)\sum_{k=1}^{n_i} x_{ij}^k, i = 1, \ldots, p; j = 1, \ldots, m$ are the sample means and $s_j^2 = (\sum_{i=1}^{p}\sum_{k=1}^{n_i}\left(x_{ij}^k - \bar{x}_{ij}\right)^2)/\nu$, $j = 1, \ldots, m$, are the pooled sample variances, with $\nu = (\sum_{i=1}^{p} n_i - p)$. The null distribution of each T_{ij}^{Dunn} is univariate t-distribution with ν degrees of freedom. The vector of Dunnett test statistics $\boldsymbol{T_j^{Dunn}} = \left(T_{1j}^{Dunn}, \ldots, T_{qj}^{Dunn}\right)$ has a q-variate t-distribution with ν degrees of

freedom and correlation matrix $\boldsymbol{R} = (\rho_{ik})_{q\times q}$, where ρ_{ik} are defined in Dunnett. The Dunnett-adjusted critical value for the two sided test for $\left\{T_{ij}^{Dunn}, i = 1, ..., q\right\}$, denoted by $u_\alpha(q,\nu)$, is the quantile of the above q-variate t-distribution such that,

$$Pr\left(\left|T_{1j}^{Dunn}\right| \leq u_\alpha, ..., \left|T_{qj}^{Dunn}\right| \leq u_\alpha\right) = 1 - \frac{\alpha}{2},$$

or equivalently,

$$Pr\left(\max_{\{i=1,\dots,q\}} \left|T_{ij}^{Dunn}\right| \leq u_\alpha\right) = 1 - \frac{\alpha}{2}.$$

The observed values of T_{ij}^{Dunn}, t_{ij}^{Dunn}, say, are compared to $u_\alpha(q,\nu)$ and we reject H_{0i}^j if $\left|t_{ij}^{Dunn}\right| > u_\alpha(q,\nu)$. For each gene j we have a vector of observed Dunnett test statistics, $\boldsymbol{t}_{ij}^{Dunn} = \left(t_{1j}^{Dunn}, \dots, t_{qj}^{Dunn}\right)$. Let, $t_j^{max} = \max_{i=1,\dots,q}\left|t_{ij}^{Dunn}\right|$. We obtain the screening P-value for testing the screening hypotheses (S2) as follows:

$$\begin{aligned} & Pr\left(\max_{\{i=1,\dots,q\}} |T_{ij}| > t_j^{max}\right) \\ = \quad & 1 - Pr\left(-t_j^{max} \leq T_{ij} \leq t_j^{max}, i = 1, \dots, q\right), \end{aligned} \tag{S11}$$

where, the probability is obtained from the CDF of q-variate t-distribution with ν degrees of freedom and the correlation structure defined in Dunnett [7]. Let R denote the number of rejected screening hypotheses while applying the BH procedure to these screening p-values.

Dunnett mdFWER controlling procedure for Steps 2-3: We use Dunnett procedure to obtain the Dunnett-adjusted p-values, $\tilde{P_{ij}}^{Dunnett}$, for testing the pairwise hypotheses as follows,

$$\tilde{P_{ij}}^{Dunnett} = 2 \cdot Pr\left(\max_{\{i=1,\dots,q\}} \left|T_{ij}^{Dunn}\right| \geq t_{ij}^{Dunn}\right). \tag{S12}$$

We reject H_{0i}^j if the corresponding adjusted P-value $\tilde{P_{ij}}^{Dunnett} \leq \frac{R\alpha}{m}$ and conclude $\theta_{ij} > 0$ if $T_{ij}^{Dunn} > 0$ and vice versa.

S4 Supplementary Results for the Simulation Study

Methods Used in Simulation Study

In this section we describe the different mdFDR controlling methodologies used in the simulation study. We develop methodologies by combining Dunnett screening procedure with four different mdFWER controlling procedures for steps 2 and 3 and compare the performance of the resulting four methodologies with Guo et al. [6] methodology in terms of mdFDR control and power.

Screening Procedure for Step 1:

Dunnett P_{screen}: The Dunnett method [7, 8, 9] is a powerful method specifically designed to test hypotheses where several treatments are compared with a common control in an unbalanced one-way layout. The multiple pairwise comparisons we

make with the FGS gene expression data fit into the framework of Dunnett test [7]. The test statistics, T_{ij}, for testing the pairwise hypotheses, are obtained as described in [7], with details given in Section S3.

Procedures for Steps 2 and 3:

Dunnett Procedure: We obtain the Dunnett-adjusted pairwise p-values [8, 9], to be used in Step 2 of the algorithm and call them $\tilde{P_{ij}}$. The details are given in section S3. The procedure rejects H_{0i}^{j} if $\tilde{P_{ij}} \leq \frac{R\alpha}{qm}$ and conclude on sign of θ_{ij} based on the sign of the test statistic T_{ij}.

Holm Procedure: We use Holm's step-down procedure at level $\frac{R\alpha}{m}$ within each significant gene. Order the pairwise p-values for each significant gene j as $P_j(1) \leq \cdots \leq P_j(q)$ with corresponding hypotheses denoted as $H_0^j(1), ..., H_0^j(q)$. Let k ($k = 1, \ldots, q$) be the maximum index such that $P_j(i) \leq \frac{R\alpha}{m(q-i+1)}$ for all $i \leq k$, then reject $H_0^j(1), ..., H_0^j(k)$, conclude on direction based on the sign of the test statistic and accept the rest of the hypotheses.

Hochberg Procedure: We use Hochberg's step-up procedure at level $\frac{R\alpha}{m}$ within each significant gene to identify significant categories. Let k ($k = 1, \ldots, q$) be the maximum index such that $P_j(k) \leq \frac{R\alpha}{m(q-k+1)}$, then reject $H^j(1), ..., H^j(k)$, conclude on direction based on the sign of the test statistic and accept the rest of the hypotheses.

Bonferroni Procedure: Bonferroni procedure is a commonly used single step multiple testing procedure that strongly controls FWER. Reject H_{0i}^{j} if $P_{ij} \leq \frac{R\alpha}{qm}$ and conclude on direction based on the sign of the test statistic T_{ij}.

Guo et al. [6] Procedure:

The procedure of Guo et al. [6] is a special case of the general testing procedure. They first obtain the p-values, $\{P_{1j}, ..., P_{qj}\}$, for testing the pairwise hypotheses in (S1) and use the Bonferroni pooling to obtain the screening p-values as follows:

$$P_{screen}^{j} = q \cdot \min \{P_{1j}, ..., P_{qj}\} .$$

Note down R, the number of significant genes. For each significant gene, use the Bonferroni procedure discussed above to identify significant pairwise differences and conclude on direction.

Results for the Simulation Study

In this section we present the results from the simulation study that consider different kinds of dependencies of the gene expressions. The dependence within genes across groups means that the gene expressions from different tumor samples are dependent but given any two genes for a sample, the expressions are independent between the two genes; as several tumor samples belong to same subject, this kind of dependence structure is valid. The dependence among genes means that the gene expressions from different genes are dependent but given any two samples for a gene, the expressions are independent; as the genes belonging to same gene set have similar activity, this kind of dependence structure is also valid in the FGS microarray data.

We define the concept of average power for the three step procedure for comparing different methodologies that control the mdFDR at the same level. Let, $\mathfrak{R}$ denote the set of indices of rejected screening hypotheses $H^j_{0screen}$ with $|\mathfrak{R}| = R$. Let, $R_j, j \in \mathfrak{R}$ denote the number of pairwise hypotheses H^j_{0i} rejected for each significant gene. Let, S denote the number of false null screening hypotheses rejected and let, $S_j, j \in \mathfrak{R}$ denote the number of false null pairwise hypotheses rejected for each discovered gene. Then, the average power for the three step procedure is defined as follows:

Definition 2: *Average Power in Three Step Procedure:* The average power is defined as the expected proportion of false null hypotheses rejected in Step 1 and Step 2 among all rejections in Step 1 and Step 2,

$$\text{Average Power} = E\left(\frac{S + \sum_{j\in\mathfrak{R}} S_j}{\max\left(R + \sum_{j\in\mathfrak{R}} R_j, 1\right)}\right). \tag{S13}$$

The different mdFDR controlling procedures that are shown in the Figures 4-6 and Figures S2-S5 are: (i) The proposed methodology (Dunnett), (ii) Dunnett screening and Holm procedure (Holm), (iii) Dunnett screening and Hochberg procedure (Hochberg), (iv) Dunnett screening and Bonferroni procedure (Bonferroni) and (v) Guo et al. [6] procedure.

Dependence within genes across groups.

In this case the components Z^s_{ij}, $i = 1, ..., p$ are dependent with $Z^s_{ij} \sim N(\mu_{ij}, 1)$ and have a common correlation $\rho = 0.2, 0.5, 0.8$. The results are summarized in Figures 5 and Figures S2-S3. All five procedures control the mdFDR at less than $\alpha = 0.05$. Once again, as in the case of independence, the proposed method gains in power compared to the other methods.

Dependence among genes.

We next considered the situation where gene expressions are dependent among genes. For this simulation, the components Z^s_{ij}, $j = 1, ..., m$ are dependent with $Z^s_{ij} \sim N(\mu_{ij}, 1)$ and have a common correlation $\rho = 0.2, 0.5, 0.8$. The results are summarized in Figure 6 and Figures S4-S5. Again, all five procedures control the mdFDR at less than $\alpha = 0.05$ and as in the case of independence, the proposed method gains in power compared to the other methods.

Author details

References

1. Benjamini, Y., Hochberg, Y.: Controlling the false discovery rate: a practical and powerful approach to multiple testing. Journal of the Royal Statistical Society: Series B **57**, 289–300 (1995)
2. Shaffer, J.P.: Control of directional errors with stagewise multiple test procedures. Annals of Statistics **8**, 1342–1347 (1980)
3. Finner, H.: Stepwise multiple test procedures and control of directional errors. Annals of Statistics **27**, 274–289 (1999)
4. Heller, R., Manduchi, E., Grant, G., Ewens, W.: A flexible two-stage procedure for identifying gene sets that are differentially expressed. Bioinformatics **25**, 929–942 (2009)
5. Benjamini, Y., Heller, R.: Screening for partial conjunction hypotheses. Biometrics **64**, 1215–1222 (2008)
6. Guo, W., Sarkar, S.K., Peddada, S.D.: Controlling false discoveries in multidimensional directional decisions, with applications to gene expression data on ordered categories. Biometrics **66**, 485–492 (2010)
7. Dunnett, C.W.: A multiple comparison procedure for comparing several treatments with a control. Journal of the American Statistical Association **50**, 1096–1121 (1955)

8. Dunnett, C.W., Tamhane, A.C.: Step-down multiple tests for comparing treatments with a control in unbalanced one-way layouts. Statistics in Medicine **10**, 939–947 (1991)
9. Dunnett, C.W., Tamhane, A.C.: A step-up multiple test procedure. Journal of the American Statistical Association **87**, 162–170 (1992)
